# Screening of *In Vivo* Activated Genes in *Enterococcus faecalis* during Insect and Mouse Infections and Growth in Urine

**DOI:** 10.1371/journal.pone.0011879

**Published:** 2010-07-29

**Authors:** Aurelie Hanin, Irina Sava, YinYin Bao, Johannes Huebner, Axel Hartke, Yanick Auffray, Nicolas Sauvageot

**Affiliations:** 1 Laboratoire de Microbiologie de l'Environnement, EA956 USC INRA2017, Université de Caen, Caen, France; 2 Division of Infection Diseases, Department of Medicine, University Medical Center, Freiburg, Germany; University of Hyderabad, India

## Abstract

*Enterococcus faecalis* is part of the commensal microbiota of humans and its main habitat is the gastrointestinal tract. Although harmless in healthy individuals, *E. faecalis* has emerged as a major cause of nosocomial infections. In order to better understand the transformation of a harmless commensal into a life-threatening pathogen, we developed a Recombination-based *In Vivo* Expression Technology for *E. faecalis*. Two R-IVET systems with different levels of sensitivity have been constructed in a *E. faecalis* V583 derivative strain and tested in the insect model *Galleria mellonella*, during growth in urine, in a mouse bacteremia and in a mouse peritonitis model. Our combined results led to the identification of 81 *in vivo* activated genes. Among them, the *ef_3196/7* operon was shown to be strongly induced in the insect host model. Deletion of this operonic structure demonstrated that this two-component system was essential to the *E. faecalis* pathogenic potential in *Galleria*. Gene *ef_0377*, induced in insect and mammalian models, has also been further analyzed and it has been demonstrated that this ankyrin-encoding gene was also involved in *E. faecalis* virulence. Thus these R-IVET screenings led to the identification of new *E. faecalis* factors implied in *in vivo* persistence and pathogenic potential of this opportunistic pathogen.

## Introduction

*Enterococcus faecalis* is a ubiquitous lactic acid bacterium and a core constituent of the intestinal flora of humans and many animals. The intrinsic ability of this bacterium to resist strongly against stressing environments may allow the bacterium to persist in hospital environments and to survive host defences [1], [2]. In the last decades, *Enterococci* have been recognized as one of the most common bacteria involved in hospital-acquired infections [3]. Indeed, these microorganisms can trigger serious infections such as sepsis, urinary tract infections, peritonitis and endocarditis and the species *E. faecalis* is still responsible for the majority of human enterococcal infections [3], [4]. For this reason, clinical strains have been studied for their virulence-associated factors: the Cytolysin CylL [5], the Aggregation substance Agg [6], the metallo-endopeptidase GelE [7], the Extracellular Surface Protein Esp [8], and the cell surface protein EfaA [9]. Despite studies characterizing these proteins, our knowledge of the mechanisms involved in infections, especially transcriptional modulation occurring in living hosts, remains incomplete. Thus, in order to further increase our understanding of enterococcal pathogenesis, it is necessary to identify genes that are specific to infection.

In an infected host, microorganisms are subjected to combined stresses. Although these conditions could be simulated *in vitro* by the study of each of these stresses independently, it is not possible to reproduce an exact mimic of the complex and dynamic environment encountered by bacteria in infected hosts. In a different approach, several *In Vivo* expression technologies (IVET) have been developed [10], [11]. With this technology, the living infected animal is the inducing-signal of virulence-associated genes expression. An IVET strategy was developed for the first time in *Salmonella enterica* serovar Typhimurium to identify *in vivo* highly expressed genes as compared to expression under laboratory conditions. Five *in vivo*-induced (*ivi*) operons have been identified using this strategy, originally based on the use of an auxotrophic marker. Three of them, corresponding to the *carAB*, *pheST-himA* and *rfb* operons, have been confirmed to play essential roles in virulence [12]. Other IVET approaches, using antibiotic resistance markers [13] or dual reporters [14]–[16], have also been employed. However, these strategies present common drawbacks due to the fact that weakly or transiently expressed *ivi* genes are difficult or impossible to detect. These disadvantages have led some authors to choose a fourth IVET approach, called R-IVET for Recombination-based IVET [17].

This molecular biology strategy functions as a genetic screening that allows the detection of the specific expression of genes *in vivo*. The method has been successfully used to identify 72 *Lactobacillus plantarum* genes induced in the gastrointestinal tract of mice [14], [17]. This approach is based on the irreversible activity of the site-specific recombinase Cre from the bacteriophage P1. Only a short or low pulse of recombinase expression is necessary to trigger a resolution leading to the permanent excision of an antibiotic marker flanked by two copies of site-specific recombination sequences (*loxP*) and hence to a phenotypic switch that enables a selection of resolved cells after recovery of bacteria from a host. Thus, R-IVET allows also the identification of transient or conditional promoter activations [17]. To this end, a genomic library of a given bacterium is created by cloning DNA fragments upstream from a promoterless copy of the recombinase-encoding gene. At the same time, the substrate for resolution is constructed by integrating the antibiotic resistance gene and the two *loxP* sites into the bacterial chromosome. These modified bacteria can then be screened under chosen conditions, for instance by inoculating them into a susceptible living host model such as mice or insects.

A R-IVET screen using the TnpR recombinase has been performed recently in *Enterococcus faecalis* OG1RF showing that R-IVET was a suitable strategy in this microorganism. This allowed the identification of 68 loci with a putative involvement in biofilm formation [18]. Some of these promoters were confirmed by quantitative RT-PCR to be induced under these conditions.

In this paper, we describe the construction of a novel R-IVET promoter-trap strategy and its application to the *Enterococcus faecalis* V19 strain, a plasmid-cured derivative of the clinical isolate V583. This is the first time that such an *in vivo* expression technology has been used in a context of virulence in Enterococci in order to study the *in vivo* behavior of this bacterium. Transcriptional activations during adaptation and response to host environment were firstly monitored after the injection of these bacteria into the hemocoel of *Galleria mellonella* larvae. This insect model has been shown to be a practical and well-adapted model for the *in vivo* screening of virulence-related genes [19]–[21]. To approach conditions encountered by these bacteria during human infections, further studies were performed during exposure to urine, in a peritonitis mouse model and in a mouse bacteremia mouse model thus leading to the identification of new genetic determinants putatively involved in the pathogenic potential of *Enterococcus faecalis*.

## Results and Discussion

### Construction of a R-IVET system in *E. faecalis*

**Figure 1:**
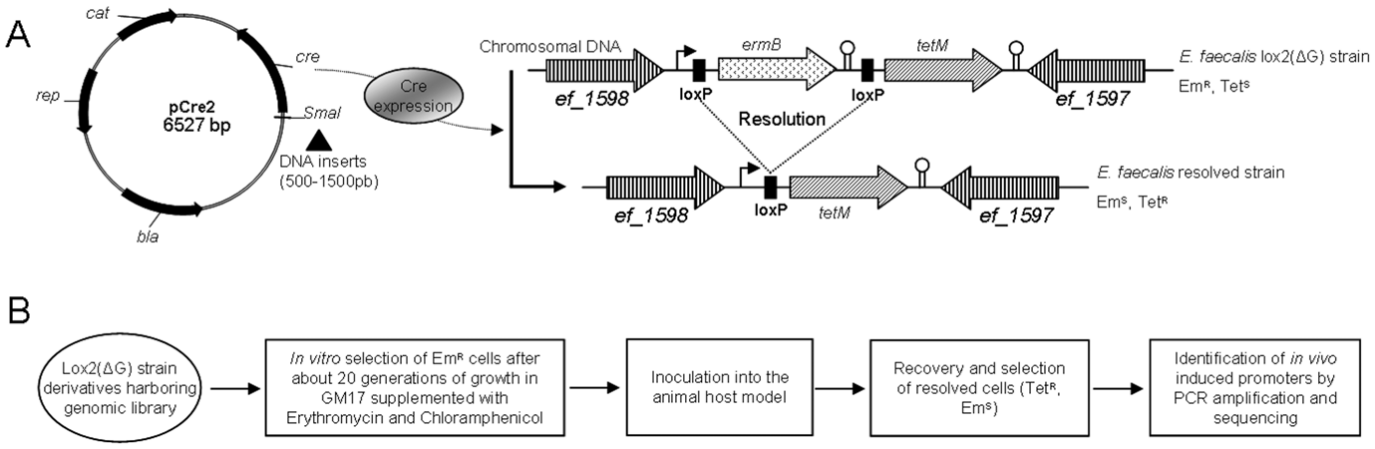
R-IVET strategy employed in *E. faecalis* to identify promoters that are induced *in vivo*. **A: Modification of Lox2 strain occurring when a promoter is active**. Chromosome of V583 strain was modified by integrating *ermB* gene and a promoterless copy of *tetM* gene, first of which is flanked by two copies of site-specific recombination sequences (*loxP*). A V583 genomic library was constructed by cloning DNA fragments in plasmid pCre2 upstream from *cre* gene. If an active promoter is thus cloned, the recombinase Cre would be expressed resulting in a homologous recombination event between both *loxP* sites. This triggers the irreversible excision of the *ermB* gene and *tetM* becomes functional. **B:** ***In vivo*** **screening of** ***E. faecalis*** **V583 genomic library**. After elimination of cells containing an active promoter under *in vitro* conditions, the resulting library is administrated to the animal host model. After infection, changes in the antibiotic resistance phenotype allow to select resolved bacteria containing an *in vivo* induced promoter.

**Figure 2:**
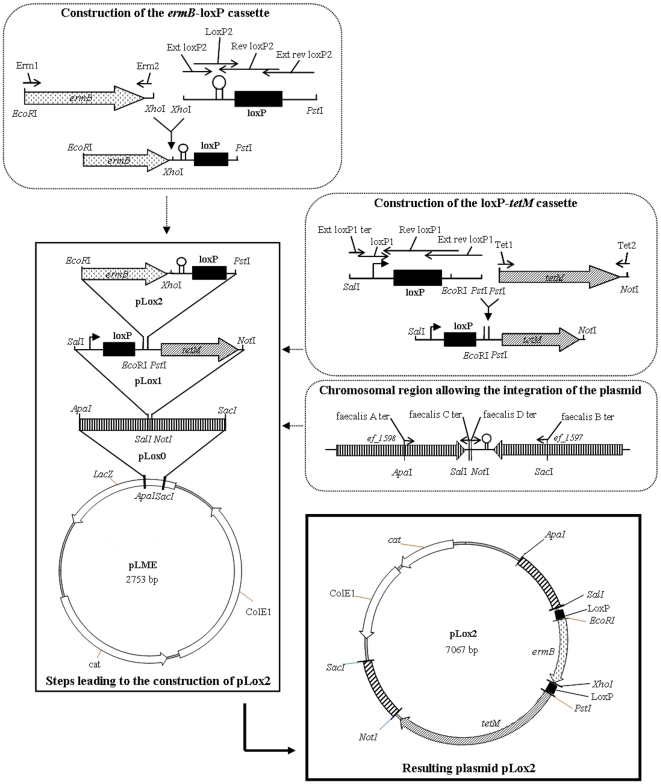
Schematic representation of cloning steps leading to the construction of the integration vector pLox2. An amplicon resulting from the amplification of a genomic fragment was first subcloned into pLME vector in order to allow the integration of the *loxP-ermB-loxP-tetM* cassette in the intergenic region between loci *ef_1597* and *ef_*1598. The resulting plasmid, pLox0, is then used to add a *loxP-tetM* cassette between these both genes. The obtained vector was named pLox1. A cassette *ermB-loxP* was finally cloned into pLox1 to obtain the integration plasmid pLox2. Only relevant restriction sites are indicated. *ColE1* = colE1 origin; *cat* = Cm^R^ encoding gene from pNZ7125 [17]; *ermB* = erythromycin rRNA methylase gene from pUCB30 [51]; *tetM* = TetR encoding gene from p3Tet [27]. Thin arrows represent primers used in these constructions.

In order to identify bacterial genes that are induced during the persistence of *E. faecalis* in an animal host, a R-IVET strategy has been performed. The system previously developed in *Lactobacillus plantarum* [17] was adapted to *E. faecalis* and improved to allow a positive selection of resolved cells by an appropriate antibiotic selection. Therefore, a *loxP*-*ermB*-*loxP*-*tetM* cassette was integrated into the chromosome of the erythromycin sensitive *E. faecalis* V19 strain (Fig. 1A). To obtain this derivative strain, some intermediate plasmids had to be constructed (Fig. 2). The *ermB* gene and a *loxP* sequence separated by a strong terminator (ΔG = −36.3kcal±10%, [22]) were cloned between the first *loxP* site and *tetM* gene in plasmid pLox1. The resulting plasmid pLox2 thus carries the full *loxP*-*ermB*-*loxP*-*tetM* cassette in which the expression of the antibiotic resistance genes is controlled by the constitutive promoter from transposon Tn1545 [23] and the presence or absence of the terminator (see materials and methods part). Plasmid pLox2 was then introduced into strain V19 and subsequently integrated into the genome of this strain by a double crossover event. The V583 derivative strain thus obtained, harboring the *loxP*-*ermB*-*loxP*-*tetM* cassette was named Lox2. Southern blot experiments confirmed that the chromosomal construction was present in a single copy between genes *ef_1597* and *ef_1598*, as expected. Our reporter system has been chosen to be integrated into this intergenic region of the *E. faecalis* chromosome in order to improve the stability of the construction. Both genes are convergent and a terminator located downstream from *ef_1597* prevents the synthesis of an antisense-RNA attenuating the expression of the antibiotic resistance genes. Moreover, expression of the deoxyribodipyrimidine photolyase (*ef_1598*) would enhance the activity of the promoter cloned upstream from the first *loxP* site.

Growth experiments in liquid GM17 or isolation of strains on agar plates containing GM17 supplemented with appropriate antibiotics confirmed the suitability of the construction in *E. faecalis*. Indeed, Lox2 strain can grow if erythromycin is present but not in the presence of tetracycline contrary to Lox1 strain. This latter strain was constructed by integrating plasmid pLox1 into the chromosome of strain V19 by a double cross-over event and used as an excision control because the integrated region in this strain is identical to that of the resolved Lox2 strain (Fig. 1A). The Lox1 strain was shown to be able to grow in the presence of tetracycline but not if erythromycin is present in the medium. The whole *loxP*-*ermB*-*loxP*-*tetM* cassette was finally sequenced.

**Figure 3:**
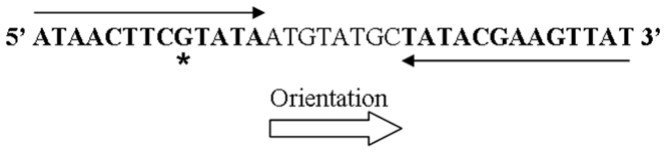
*LoxP* sequence. *LoxP* sites are composed of two 13 pb inverted sequences separated by an 8 pb spacer region which gives the orientation of the *LoxP* site [56]. The base indicated by * is deleted in the second *loxP* site of Lox2ΔG strain.

In order to allow us to identify promoters that are weakly expressed under *in vitro* conditions but that are strongly activated *in vivo*, we decided to use also another strain that contained a 1 base-deletion in the second *loxP* site, as shown in Fig. 3. This strategy has already been used and it has been demonstrated that mutations in *loxP* reduce the affinity of the enzyme Cre for these sites thus decreasing the level of sensitivity of the reporter system [24]. Both strains, Lox2 (without mutation) and Lox2ΔG (with mutation) were used in this study thus providing a R-IVET system with two different levels of sensitivity.

### Construction of a *E. faecalis* V583 genomic library in Lox2(ΔG) strains

To generate the V583 genomic library, the replicative vector pCre2 has been used. This plasmid contains a *cat* gene, conferring resistance to chloramphenicol, and a promoterless copy of the *cre* gene, encoding the site-specific recombinase Cre. *AluI*-digested *E. faecalis* genomic fragments were cloned upstream from *cre* gene using *E. coli* VE14188 as an intermediate host. PCre2 derivatives thus obtained from 57,600 *E.coli* colonies were extracted to be introduced in Lox2 and Lox2ΔG strains. 58,000 independent clones were recovered with Lox2 strain. PCR analysis of 90 clones suggested that 68% of them contained inserts with an average size of 1.1 kb. In the Lox2ΔG strain, about 85,000 clones were recovered of which, 74% had an insert with an average size of 900 pb. These results showed that the libraries covered in both strains more than 99% of the *E. faecalis* V583 genome. To evaluate the libraries redundancy, 22 inserts were sequenced suggesting that libraries are random, not redundant and that no specific genomic region was over- or underrepresented. It can also be noticed that fragments of plasmids pTEF1 and pTEF2 were represented in the libraries. The percentage of clones active under laboratory conditions was also evaluated by calculating the resolution frequency in the library before subcultures in an erythromycin-containing medium. It was thus shown that 8% of clones were resolved suggesting that 8% of the library inserts contained active promoters.

### R-IVET screen in the *Galleria mellonella* host model

Several R-IVET screenings have been performed in this study in order to identify genes involved in *E. faecalis* pathogenesis. First we used a surrogate virulence model based on the insect *G. mellonella*. This model is increasingly used since the immune system of the larvae used for infection exhibits a high degree of structural and functional similarity with the innate immune system of mammals [19], [21]. Recently it has been demonstrated that this model is also useful to evaluate virulence of *E. faecalis* [25], [26].

Because this approach aims to highlight promoters that are specifically activated during infection in comparison with *in vitro* growth conditions, the first step consisted in eliminating cells in which promoters that are active under laboratory conditions were cloned upstream from *cre* gene. These pCre2 derivatives were removed from screening by subculturing R-IVET libraries for 20 generations on GM17 supplemented with erythromycin and cells were plated on an agar medium containing tetracycline (Fig. 1B). No resolved clone was observed after this subculturing step in the Lox2ΔG library, but it was noticed that a percentage of clones from the Lox2 library was spontaneously resolved (reaching 0.12%±0.12 clones). This rate of spontaneous excision in the Lox2 library called into question the reliability of our R-IVET system. Tet^R^ and Em^S^ colonies from the Lox2 library were used as a template for PCR amplification of the insert cloned upstream from *cre* and amplicons were finally used for sequencing. Interestingly, upon more than 50 spontaneously resolved colonies analyzed, always the same three inserts have been found. Indeed, clones containing the promoters of *ef_0020*, *ef_0092* (and *ef_0093*) and *ef_0573* became Em^S^ and Tet^R^ at any time even though any stress had already been applied to bacteria. Therefore these genes were to remove from results and from further studies because of their non-specificity to *in vivo* conditions.

**Figure 4:**
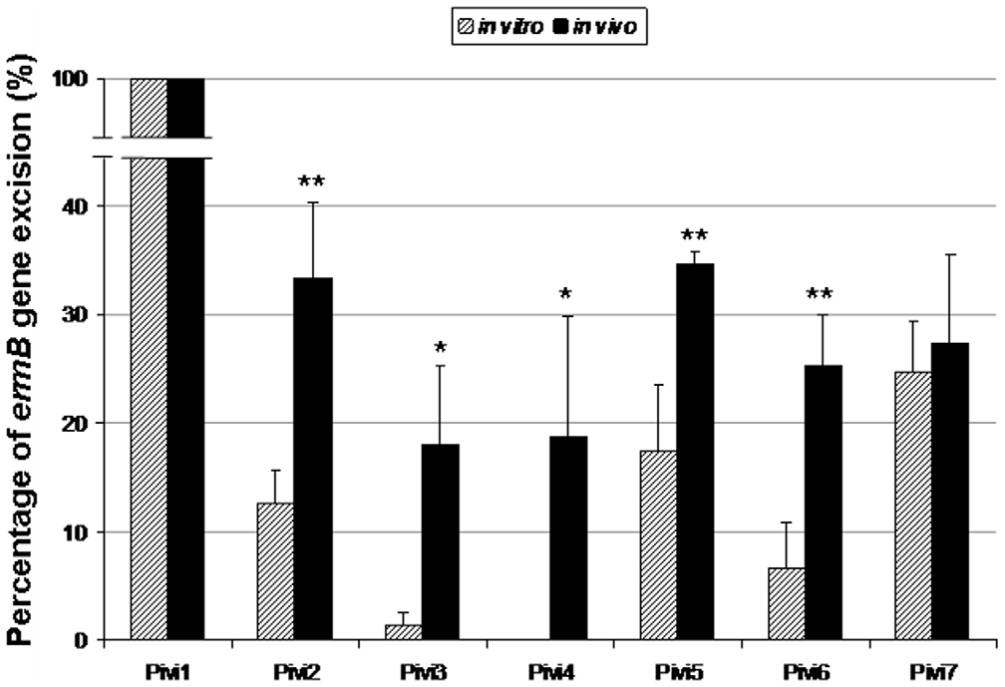
Individual resolution analysis of R-IVET identified promoters. Plasmids containing a putative *in vivo* induced promoter cloned upstream from *cre* gene were extracted from resolved strain to be introduced in the unresolved Lox2 (or Lox2ΔG) strain. Resolution rate was measured after 15 generations of *in vitro* growth or after *in vivo* persistence by calculating the percentage of Em^S^, Cm^R^ and Tet^R^ CFU. Values correspond to the mean of three measurements obtained after independent experiments. Error bars indicate standard deviations. A Student test was used to determine the following P values when comparing *in vitro* resolution to *in vivo* induction: for Pivi1, nd; for Pivi2, 0.009; for Pivi3, 0.017; for Pivi4, 0.043; for Pivi5, 0.008; for Pivi6, 0.007; and for Pivi7, 0.646. All promoters indicated by an asterisk are significantly induced during *in vivo* persistence (*, p≤0.05; **, p≤0.001).

**Figure 5:**
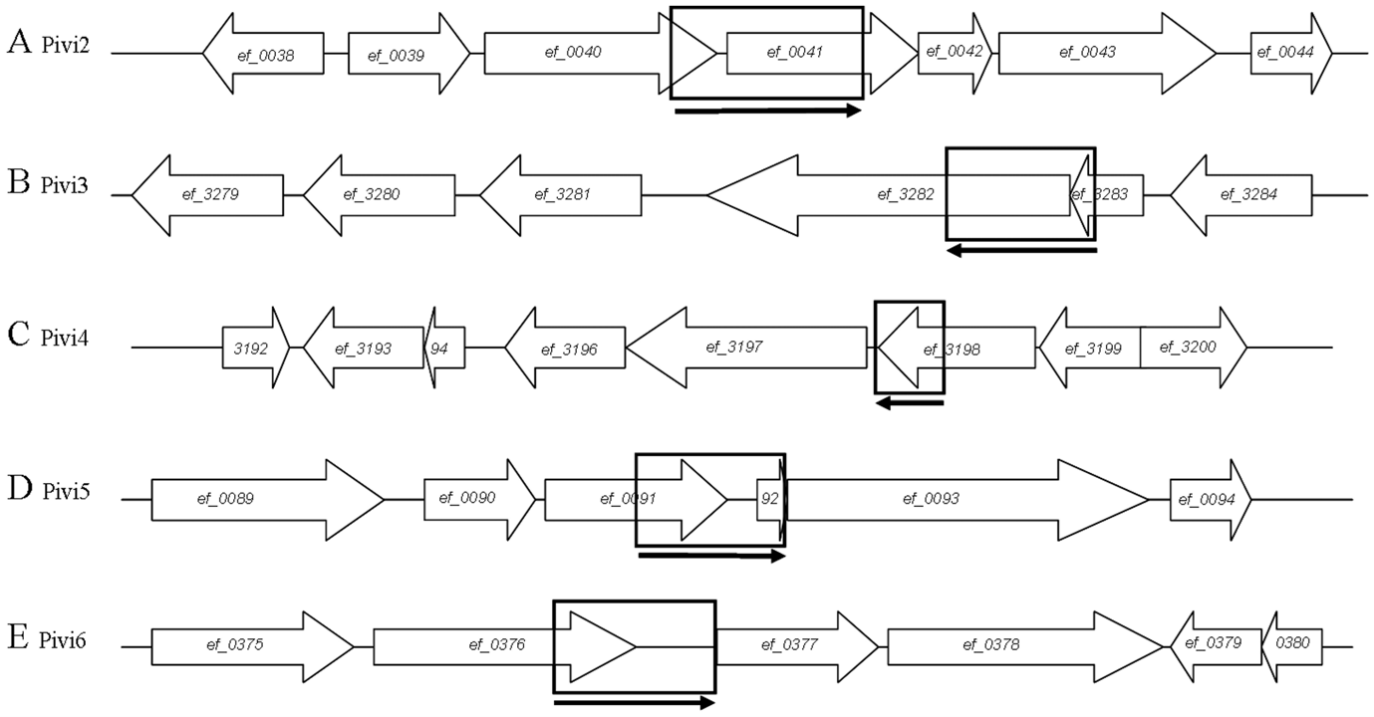
Locations of *in vivo* induced promoters on *E. faecalis* chromosome. Promoters that were shown to be induced during persistence in *Galleria mellonella* larvae are located in open boxes and arrows below the boxes indicate the orientation in which promoters were cloned upstream from the recombinase gene in plasmid pCre2. Thick arrows represent Open Reading Frames located in the region neighboring these promoters. **A**: Pivi2 is located within a 1.2 kb-long region overlapping *ef_0040*, *ef_0041* and the untranslated region between them. Pivi2 probably regulates *ef_0041* expression. **B**: Pivi3 is located within a 1.1 kb-long fragment overlapping *ef_3282* and *ef_3283* and should control *ef_3282* expression. **C**: Pivi4 is included in a 550 pb-long region overlapping *ef_3198* and a small part of untranslated region between *ef_3197* and *ef_3198*. This suggests that Pivi4 regulates the expression of the two-component system encoding operon *ef_3196/7*. **D**: Pivi5 is located in a 1.2 kb-long fragment overlapping *ef_0091*, *ef_0092* and the untranslated region between them. Pivi5 could regulate *ef_0092* and/or *ef_0093* expression. **E**: Pivi6 is included in a 620 pb-long region overlapping *ef_0376* and the untranslated fragment between this gene and *ef_0377*. So Pivi6 could control expression of *ef_0377* and *ef_0378* which seem to be co-transcribed.

Both libraries (Lox2 and Lox2ΔG) were then screened in the *G. mellonella* insect model. The infected larvae were incubated at 37°C for 16 hours. Melanized larvae were sacrificed, haemolymph was recovered, diluted and plated. Replica plating of colonies then allowed the selection of bacteria in which a promoter cloned upstream from *cre* was activated during persistence in the insect host. Indeed, clones harboring a pCre2 derivative in which a promoter was activated at any time during passage in the host model are able to grow on plates containing tetracycline and chloramphenicol but not erythromycin (Fig. 1B). This resulted in the identification of 16 Lox2 resolved clones and 3 Lox2ΔG resolved clones, respectively corresponding to 0.74 and 0.26% of the screened colonies. According to the genome annotation database, only one of the inserts did not contain an untranslated region upstream from a coding sequence whereas the 18 other candidates corresponded to 7 redundant putative promoter regions. These promoters were checked for *in vivo* induction by individual screening *in vitro* and in the insect host. Plasmid pCre2 derivatives containing a putative *in vivo* induced promoter were extracted from resolved Em^S^ and Tet^R^ cells before being introduced into the unresolved Em^R^ and Tet^S^ Lox2ΔG or Lox2 strains. To compare expression of putative promoters in *in vitro* conditions and during infection in *G. mellonella*, resolution frequencies of the reporter cassette were measured by determining the rate of *ermB* gene excision as described in the experimental procedures (Fig. 4). The resolution rate was measured for each putative *in vivo* induced promoter. For 5 of the 7 clones, promoters were thus subsequently confirmed to be significantly induced in the insect host model, with an *in vivo* resolution at least twice greater than *in vitro* resolution. This suggests that the corresponding genes are important for the infection process and hence for virulence. The genomic locations of these promoting regions were mapped by comparing insert sequences to the genomic sequence of *E. faecalis* V583 (Fig. 5). The genetic organization of three regions (Pivi4, 5 and 6) suggests that the identified promoters could control the expression of bicistronic operons. According to the control experiments described above, the promoter of the operon *ef0092/3* which corresponded to Pivi5 was removed from results and the genes controlled by the other identified promoters are listed in Table 1. Functions of three of these genes remain unknown but the three others are involved in protein degradation or signal transduction.

**Table 1:** *E. faecalis* genes induced during persistence in the *Galleria mellonella* host model.

N°	ORF^a^	Annotation^b^	Cellular role^b^
Pivi2	*ef_0041*	PIN domain protein	Unknown function
Pivi3	*ef_3282*	ATP-binding subunit ClpC	Protein fate
Pivi4	*ef_3196*	Response regulator	Signal transduction
	*ef_3197*	Sensor histidine kinase	Signal transduction
Pivi6ΔG	*ef_0377*	Ankyrin repeat protein	Unknown function
	*ef_0378*	N-acyl-D-amino-acid deacylase family protein	Unkonwn function

a Promoter region mapping led to the identification of genes putatively controlled by *in vivo* induced promoters.

b Annotation and cellular role category of genes were determined as specified in TIGR genome database.

ΔG Genes identified in Lox2ΔG library.

As a control, plasmid pCre2 without any insert cloned upstream from *cre* was introduced into Lox2 and Lox2ΔG strains in order to confirm that induction is not due to the presence of the plasmid. No resolution was detected before or after infection for the Lox2ΔG:pCre2 strain and resolution rate was not significantly different before and after the larvae infection (0.56%±0.97 and 1.55%±2.56, respectively) for the Lox2:pCre2 strain confirming that the promoter activation is necessary for *ermB* gene excision.

Even if the spontaneous excision rate of the Lox2:pCre2 strain remains unexplained, it was shown that the vast majority of identified promoters were significantly induced *in vivo* thus confirming the suitability of our R-IVET system in *E. faecalis*.

### Involvement of an *in vivo* induced two-component system in the pathogenic potential of *E. faecalis* in the *Galleria mellonella* infection model

**Figure 6:**
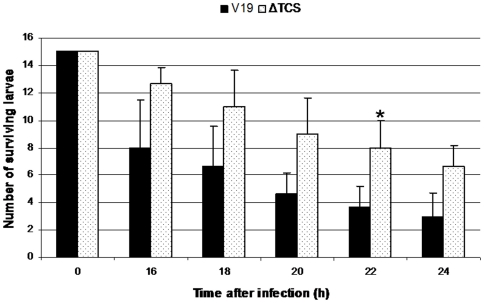
Effect of the deletion of the *ef_3196/7* operon on virulence. Deletion mutant of the *in vivo* induced genes encoding a two-component system (*ef_3196/7* operon) has been constructed. Surviving *G. mellonella* larvae are counted from 16 to 24 hours after infection with *E. faecalis* V19 (black bar) and *E. faecalis* ΔTCS (white bar). Bars indicated by an asterisk show a significant difference in comparison with wild type (*, p≤0.05). Experiments were repeated at least three times.

Independent individual analysis of each promoter has shown that Pivi4 was not active under *in vitro* conditions whereas it was strongly induced during insect infection (Fig. 4). Thus transcriptional activation controlled by Pivi4 seems to be specific to *in vivo* persistence suggesting that the two-component system regulated by Pivi4 could be essential to the pathogenic potential of *E. faecalis*. To confirm this hypothesis, a deletion mutant of this operon, named ΔTCS, was constructed and used to evaluate its virulence in comparison to the wild type strain in the *G. mellonella* model (Fig. 6). The number of surviving larvae was followed from 16 to 24 hours after infection. The results showed that the ΔTCS mutant was significantly less virulent than the wild type. We conducted this experiment also with the insertional mutant RR02 used in a previous study [27]. Similar results as with the ΔTCS mutant have been obtained. These experiments demonstrated that *ef_3196/7* operon seems indeed to be implicated in virulence confirming that R-IVET is a valuable tool to identify new fitness/virulence factors in *E. faecalis*. Of note, the previous work by Hancock and Perego did not identify a significant phenotype neither in stressing environments not in biofilm formation of the RR02 insertional mutant [27].

### R-IVET screening during exposure to urine

To identify genes specifically activated during urinary tract infections, R-IVET libraries were screened during a 3 hours challenge in urine, as described in experimental procedures. Whereas no resolved colony has been obtained with the Lox2ΔG library, 18 Cm^R^, Em^S^ and Tet^R^ colonies (1.01% of the screened bacteria) were identified from Lox2 library. Sequencing revealed that 3 of them did not correspond to obvious promoting regions. The 15 remaining sequences corresponded to 12 loci containing one or several promoters controlling a total of 17 ORFs. Genes controlled by these specifically *in vivo* induced promoters are listed in Table 2. Two inserts contained two *AluI* restriction fragments (Uivi7 and Uivi8) and harbored two putative promoting regions. Concerning Uivi7, one of the two fragments contained the putative promoter of *ef_0839* or *ef_2975* (these both genes are strictly identical). Even though the functions of the majority of these genes remain unknown, it can be noticed that the other genes are involved in transport or binding to substrates and cell envelope composition. Quantitative RT-PCR (qRT-PCR) was then used to confirm that clones, identified in the R-IVET screening, contained promoters that were significantly activated during exposure to urine compared to growth in a rich medium. For this experiment, RNAs were extracted from *E. faecalis* V19 cells after 3 hours of exposure to filter-sterilized urine or in GM17 under the same conditions used for the R-IVET screening. Expression levels were measured for 17 genes and the expression of *ef_2959*, *ef_2960*, *ef_3324* and *ef_3325*, encoding a putative ribose uptake protein, a ribose transporter, the beta-subunit and the biotin carboxyl carrier of a sodium ion-translocating decarboxylase, respectively, was shown to be expressed at a higher level at 3 hours of exposure to urine (induction factors were 13.1±5.2, 4.3±1.9, 5.6±4.4 and 12.9±1.5 respectively). This suggests that bacteria overexpress new transport pathways in order to adapt to nutritional conditions encountered in urine. Since qRT-PCR allows the quantification of a given transcript at a defined time (in this case, 3 hours), the other genes identified by R-IVET screening and not confirmed by qRT-PCR might be induced at another moment during urine exposure. An analysis of the transcriptome of *E. faecalis* V583 in response to growth in blood has recently been performed [28]. Cluster of citrate catabolism, including *ef_3324* and *ef_3325*, has been demonstrated to be up-regulated in response to blood exposure. Both transcriptional studies, in blood and in urine, suggest that these genes are important for *E. faecalis* to grow in biofluids.

**Table 2:** *E. faecalis* genes induced during a challenge in urine.

N°	ORF^a^	Annotation^b^	Cellular role category^b^
Uivi2	*ef_2959*	Ribose uptake protein, putative	Transport and binding proteins
	*ef_2960*	Ribose transporter protein RbsD	Transport and binding proteins
Uivi3	*ef_3324*	Sodium ion-translocating decarboxylase, Beta subunit	Transport and binding proteins, Energy metabolism
	*ef_3325*	Sodium ion-translocating decarboxylase, Biotin carboxyl carrier protein	Transport and binding proteins, Energy metabolism
Uivi4	*ef_0153*	Cell wall surface anchor protein	Cell envelope
	*ef_0154*	Conserved hypothetical protein	Unknown function
Uivi5	*ef_0330*	SNF2 domain protein	Unknown function
Uivi6	*ef_0418*	ABC transporter, ATP-binding protein	Transport and binding proteins
Uivi7	*ef_0839*	Conserved hypothetical protein	Unknown function
	*ef_1434*	DnaD domain protein	Unknown function
	*ef_2975*	Conserved hypothetical protein	Unknown function
Uivi8	*ef_0528*	Cytolysin B transporter protein, truncation	Toxin production and resistance, Protein fate
	*ef_3251*	Hypothetical protein	Unknown function
Uivi10	*ef_0175*	Cytidine deaminase cdd	Salvage of nucleosides and nucleotides
	*ef_0176*	Basic membrane family protein	Cell envelope
Uivi11	*ef_2987*	Conserved hypothetical protein	Unknown function
	*ef_2988*	Conserved hypothetical protein	Unknown function
Uivi12	*ef_1201*	Conserved hypothetical protein	Unknown function

a Insert identification and comparison to the *E. faecalis* V583 genome database.

b Annotation and cellular role category of genes were determined as specified in TIGR genome database.

### Highlighting of *E. faecalis* genes activated during bacteremia and peritonitis infections

Next we were interested in identifying genetic determinants that are activated during bacteremia and peritonitis. Therefore, 5×10^8^ CFU and 1.7×10^10^ CFU of Lox2 or Lox2ΔG libraries were injected intravenously via the tail vein and into the peritoneal cavity of 8 BALB/c mice, respectively. In the case of bacteremia, 2, 4, 6 and 24 hours after inoculation, mice were sacrificed, blood and kidneys were obtained, homogenized and plated on a tetracycline containing medium. Four and 2 Em^S^, Tet^R^ and Cm^R^ clones were obtained from blood recovered 2 and 4 hours after inoculation, respectively. After 6 and 24 hours, no bacteria could be cultured from the blood. However, additional clones were isolated from kidneys of these animals. Indeed, a total of 50 Em^S^, Tet^R^ and Cm^R^ colonies was recovered after 2, 4, 6 or 24 hours of infection. In the peritonitis model, the relatively high number of inoculated bacteria resulted in 280, 219 and 265 Em^S^, Tet^R^ and Cm^R^ colonies that were recovered from blood, kidneys and after a peritoneal lavage, respectively. Because of the high concentration of bacteria inoculated into animals, the experiment was conducted for only 6 hours to avoid death of the animals during the experiment. As described above, resolved colonies were used as a template for PCR amplification of the insert cloned in plasmid pCre2 upstream from *cre* gene. Both R-IVET screenings using the bacteremia and peritonitis mouse models led to the identification of 64 putative genes that are activated during the pathogenic process. These *in vivo* induced genes were functionally listed in genes involved in nutrient absorption and metabolism (23 ORFs), cell wall composition (4 ORFs), extracellular functions (1 ORF), transport (4 ORFs), regulation (7 ORFs) and other functions (6 ORFs). The 19 remaining genes encode hypothetical proteins (Table S1). As already observed in previous studies [17], [20], [29], a large part of these ORFs are involved in metabolism and transport. This suggests that microorganisms have to adapt to the *in vivo* nutrient specific conditions. Moreover, 7 transcriptional regulators have been identified, thus indicating that bacteria develop a specific and adaptive response to its host-associated environment.

### Identification of *ef_0377* as a gene induced during *in vivo* persistence in insect and mice infection models and involved in the pathogenesis of *E. faecalis*

**Figure 7:**
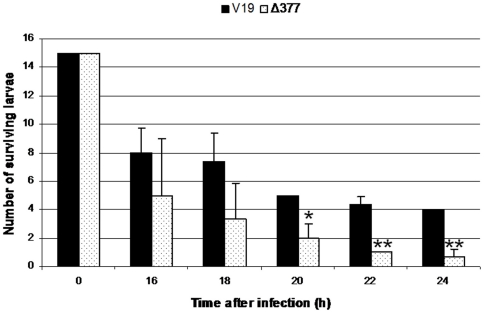
Effect of the deletion of the *ef_0377* gene on virulence. Deletion mutant of the *in vivo* induced genes encoding an ankyrin repeat protein (*ef_0377*) has been constructed. Surviving insects are counted from 16 to 24 hours after infection with *E. faecalis* V19 (black bar) and *E. faecalis* Δ377 (white bar). Bars indicated by an asterisk show a significant difference in comparison with wild type (*, p≤0.05; **, p≤0.01). Experiments were repeated at least three times.

Lists of induced genes resulting from the infection models were compared, thus highlighting 21 ORFs whose expressions were found to be activated in at least two different conditions (Table 3). One of these genes was chosen for further investigations. A promoter controlling the expression of *ef_0377*, encoding an ankyrin repeat protein, had been demonstrated to be strongly induced in the hemocoel of *G. mellonella* larvae and had also been identified in the peritonitis mouse model. In some Gram-negative pathogens, ankyrin-like proteins are involved in intracellular proliferation or persistence within macrophages [30], [31] and in oxidative stress response [32]. From these combined results, we suggested that *ef_0377* could be involved in *E. faecalis* pathogenicity. To test this hypothesis, a deletion mutant was constructed in *E. faecalis* strain V19. This strain, named Δ377, was used to evaluate its virulence in the *G. mellonella* model. Obtained results showed that Δ377 mutant was somewhat more virulent than the wild type (Fig. 7). Similar results have been obtained with an insertional 377::Ery mutant. These results suggest that the ankyrin protein encoded by this gene could be essential to the bacterial *in vivo* persistence or the host colonization even though its expression affects the virulence potential of *E. faecalis*. This has already been observed by Hollands *et al* in *Streptococcus* in which a mutation in the CovRS two-component system enhances the pathogenic potential of the bacteria whereas it reduces its ability to colonize the host [33].

**Table 3:** *E. faecalis* genes induced in at least two different conditions^a^ as compared to *in vitro* growth.

Locus	Annotation		Insect	Bacteremia	Peritonitis	Urine
*ef_0041*	PIN domain protein		x		x	
*ef_0104*	Arginine deiminase, ArcA				x	
*ef_0105*	Ornithine carbamoyltransferase, ArcF-1				x	
*ef_0107*	Transcriptional regulator Crp/Fnr, ArcR		x		x	
*ef_0108*	C4 dicarboxylate transporter putative, ArcD		x		x	
*ef_0175*	Cytidine deaminase, Cdd				x	x
*ef_0176*	Basic membrane family protein				x	x
*ef_0185*	Phosphopentomutase, DeoB			x	x	
*ef_0186*	Purine nucleoside phosphorylase, DeoD-1			x	x	
*ef_0349*	Tail protein			x	x	
*ef_0377*	Ankyrin repeat protein		x		x	
*ef_0378*	N-acyl-D-amino-acid deacylase family protein		x		x	
*ef_0528*	Cytolysin B transport protein, truncation				x	x
*ef_1072*	Operon galactose repressor, GalR			x	x	
*ef_2405*	Hypothetical protein				x	
*ef_2424*	Pyrroline-5-carboxylate reductase, putative				x	
*ef_2987*	Conserved hypothetical protein				x	x
*ef_2988*	Rhodanese family protein				x	x
*ef_2996*	Conserved hypothetical protein				x	
*ef_3060*	Putative secreted lipase				x	
*ef_3251*	Hypothetical protein				x	x
*ef_3282*	ATP-binding subunit ClpC, protease		x		x	

a In mouse infection models, resolved cells were recovered from blood, kidneys (and peritoneal lavage fluid), 2, 4, 6 (or 24) hours after infection.

Our R-IVET reporter system was based on two strains, one of which contained a single deletion in the first *loxP* site. This would allow trapping of two types of promoters. Indeed, in the strain without mutation, the expression threshold necessary to excision is very low. Thus the identified promoters have to be not expressed at all under *in vitro* growth conditions to not being eliminated during pre-cultures. However even a weak activation of their expression *in vivo* enables their identification. On the other hand, the mutant strain allows the trapping of promoters that are not or weakly expressed *in vitro* but strongly induced *in vivo*. The combination of both systems gives a powerful tool to identify gene activation. However, our results also show that the vast majority of genes were identified within the Lox2 strain suggesting that the recombination activity was too weak in the Lox2ΔG strain to be fully efficient. Nevertheless, *ef_0377* has been shown to be activated via both Lox2ΔG and Lox2 libraries in two infection models. This demonstrates that *ef_0377* is probably weakly expressed *in vitro* and strongly induced *in vivo*.

### Confirmation of previously identified *in vivo* expressed genes by our R-IVET screens

Combined R-IVET results led to the identification of genes that had already been described as *in vivo* induced genes. Indeed, *ef_0104* [34], [29] and *ef_0105* [20], both involved in arginine catabolism, are induced in *Vibrio cholerae* or *Bacillus cereus* during intestinal infection of mice and during an oral infection in insects, respectively. The ClpC encoding gene, *ef_3282*, had also been identified by R-IVET screenings in *E. faecalis* during biofilm formation [18], in *Lb. plantarum* in the gastrointestinal tract of mice [17] and in *Bacillus cereus* in insects [20]. Moreover, it has been shown that ClpC is required for growth of *Streptococcus pneumoniae* in the lung and blood in a murine pneumonia model [35]. This ATP-binding protease plays a role in autolysis and adherence to human cells [36], [37] thus being essential for pathogenesis [38].

### *In vivo* regulation of the Pathogenicity Island of *E. faecalis* V583

Interestingly, the analysis of results in their entirety revealed that only a few number of ORFs from the PAI of *E. faecalis* were demonstrated to be up-regulated (*ef_0486*, *ef_0502*, *ef_0528* and *ef_0530*). However, gene *ef_0528 (cylB)*, encoding the cytolysin B transport protein, was found to be activated in two different models, suggesting that *cyl* operon, contributing to enterococcal virulence [39]–[41], could be induced in response to the host environment. The 3′-end of *ef_0528* and genes encoding *cylA*, and *cylI*, are missing in *E. faecalis* V583 strain [42]. However, the two-component regulatory system encoded by *cylR1* and *cylR2* genes modulating the autoinduction of the expression of this operon in response to a quorum-sensing [43] is present in this strain. A previous study demonstrated that the expression of the *cyl* operon was induced in urine during stationnary phase [44]. In these conditions, it was shown that abundances of mRNAs encoding CylM and CylB seem to evolve differently from each other. The author suggested that this was due to a difference in stability of different segments of the polycistronic transcript. However, according to our R-IVET results, we alternatively propose the presence of a *cylM* internal promoter responsible for a conditional modulation of expression of *cylB in vivo* or in biofluids. Another gene potentially induced during peritonitis is *ef_0530* encoding an AraC-type regulator. In a recent study, it was shown that this gene is involved in biofilm formation, survival within macrophages and virulence in a mouse peritoneal infection model [45]. Thus, this last finding is in perfect accordance with our R-IVET results again demonstrating the usefulness of our tool. However, even though the vast majority of sequenced fragments cloned upstream from *cre* gene corresponded to obvious promoters, some inserts contained intragenic fragments or were obviously cloned in an opposite orientation. These fragments might encode small non-coding RNAs or antisense-RNAs, suggesting that our R-IVET reporter system may also be a useful and convenient tool to identify the expression of these regulatory RNAs. Work is in progress to examine this possibility.

## Materials and Methods

### Bacterial strains and growth conditions

Bacterial strains and plasmids used in this study are listed in Table S2. *E. faecalis* strains were cultivated at 37°C without shaking in M17 medium [46] supplemented with 0.5% glucose (w/vol) (GM17) and with erythromycin (150 µg.ml-1), tetracycline (10 µg.ml-1) or chloramphenicol (15 µg.ml-1) if needed. *E. coli* strains were grown at 37°C under vigorous agitation in LB medium [47] with ampicillin (100 µg.ml-1), erythromycin (150 µg.ml-1) or tetracycline (10 µg.ml-1) when required.

### General molecular methods and sequence analysis

Restriction endonucleases, shrimp alkaline phosphatase and T4 DNA ligase were obtained from GE Healthcare and Promega, and used according to the manufacturers' instructions. PCR was performed with the thermocycler “Master Cycler Gradient” (Eppendorf, Hambourg, Germany) using GoTaq DNA polymerase (Promega, Madison, WI, USA) or “Triple Master Mix” (Eppendorf). Primers are listed in Table S2. If needed, PCR products were purified using the Nucleospin® Extract II kit (Macherey-Nagel, Düren, Germany). *E. coli* and *E. faecalis* were transformed by electroporation with the Gene Pulser Apparatus (Bio-Rad), as described by Dower *et al* [48] and Holo & Nes [49], respectively. Plasmids were extracted from *E. coli* by using the Nucleospin® Plasmid kit (Macherey-Nagel). Plasmids in *E. faecalis* were extracted as previously described [50].

### Construction of a chromosomal reporter *E. faecalis* strain of *cre* expression

The non-encoding region flanked by *ef_1598* and *ef_1597* encoding a deoxyribodipyrimidine photolyase and the catalase KatA, respectively, has been chosen to integrate the *loxP-ermB-loxP-tetM* cassette into the chromosome of *E. faecalis*. First, this region was amplified by using genomic DNA of *E. faecalis* V583 as a template with two primers combinations faecalis A ter and faecalis C ter, and faecalis B ter and faecalis D ter, thus introducing *ApaI* and *SalI*, and *SacI* and *NotI* restriction sites, respectively. A mix of both amplicons was then used as a template to amplify the whole region with the primers faecalis A ter and faecalis B ter. The resulting PCR product is thus flanked by *ApaI* and *SacI* restriction sites and includes *SalI* and *NotI* sites in its middle. This amplicon was digested by *ApaI* and *SacI* and cloned into a dephosphorylated and similarly digested plasmid, pLME (vector constructed by replacing the *bla* gene of pBlueScript 2 SK+ (Stratagene, La Jolla, US) with a *cat* gene amplified from pNZ7125 [17], data not shown). The resulting plasmid was designated pLox0. The *LoxP*1 cassette includes a strongly active promoter [23] followed by a *loxP* site. This cassette was constructed by annealing and ligating the oligonucleotides ext lox P1 ter, ext rev lox P1, lox P1 and rev *loxP*1. P3Tet [27] was used as template to amplify the gene *tetM*. This antibiotic resistance marker and *loxP*1 cassette share the same *PstI* restriction site allowing us to join them after restriction, ligation and amplification. The resulting amplicon digested by *SalI* and *NotI* was then subcloned in the similarly digested and dephosphorylated pLox0 to obtain a new plasmid named pLox1. *LoxP*2 cassette was constructed by annealing and ligating the primers ext *loxP*2, *loxP*2, rev *loxP*2 and ext rev *loxP*2 thus introducing a second *loxP* site and a strong terminator (ΔG = −36.3kcal±10%)[22]. Gene *ermB* was amplified from pUCB30 [51] and ligated to *loxP*2 cassette via a *XhoI* restriction site. The ligation product was then amplified and subcloned into pLox1 between *EcoRI* and *PstI* sites. The resulting plasmid was designated pLox2 and includes a *loxP-ermB-loxP-tetM* reporter cassette flanked by two fragments of *E. faecalis* chromosome allowing the integration of the cassette into the bacterial genome via a double crossover event.

The integrative vector pLox2 was introduced in *E. faecalis* V19 strain and integrants were first selected on GM17 plates containing 150 µg/ml erythromycin. Integrants were checked by PCR before being subcultured in GM17 without erythromycin. After about 30 generations of growth, replica plating allowed the identification of a few colonies which were both sensitive to chloramphenicol and resistant to erythromycin. The double crossover event was confirmed by PCR and Southern blotting. The resulting strain containing the *loxP-ermB-loxP-tetM* construction between loci *ef_1597* and *ef_1598* was designated *E. faecalis* Lox2.

### Construction of the *E. faecalis* genomic library

Total genomic DNA was extracted from a late log-phase culture of *E. faecalis* V583. Pelleted cells were treated with lysozyme (5 mg/ml) and RNAse A (80 µg/ml) at 37°C for 10 minutes and with a solution of Proteinase K (20 mg/ml) containing Sodium Dodecyl Sulfate (5 mg/ml) at 60°C. Nucleic acids are then purified by phenol-chloroform extraction. A sufficient amount of partially digested genomic DNA from *E. faecalis* strain V583 was obtained by repeating the digestion of 2.5 µg DNA with 0.375 units of *AluI* for 1 h at 37°C. This condition provides a maximum amount of fragments ranging from 0.5 to 1.5 kb in size. Purified DNA fragments were ligated to *SmaI* digested and dephosphorylated pCre2. This vector was constructed by adding the gene *cre* amplified from pNZ7125 [17] into plasmid pCU1 and replacing the *BglII* restriction site with a *SmaI* site upstream from *cre*. The resulting plasmids were introduced into *E. coli* VE14188. Transformants were plated on LB agar plates with ampicillin and incubated at 37°C. The 57,600 obtained colonies were collectively resuspended in LB. Plasmids were extracted from these cells to be introduced into Lox2 and Lox2ΔG strains. The approximative 85,000 (in Lox2ΔG strain) and 58,000 (in Lox2 strain) resulting colonies were resuspended in GM17 containing 15% glycerol (w/vol) and stored in aliquots at −80°C.

### Screening of the genomic library in the insect host model

To counter-select against clones in Lox2 and Lox2ΔG libraries that harbor pCre2 derivatives containing a promoter active during *in vitro* growth, the libraries were sub-cultured for about 20 generations in GM17 supplemented with chloramphenicol and erythromycin. Infection of *G. mellonella* larvae with *E. faecalis* was then performed as previously described by Park *et al*. [7]. Using a syringe pump (KD Scientific, Holliston, MA, USA), larvae (about 0.3 g and 3 cm in length) were infected subcutaneously with washed *E. faecalis* strains from over-night culture in GM17 (about 3.5×10^6^ CFU per larvae) in 10 µl of sterile saline buffer using a sterilized micro syringe, and incubated at 37°C. Sixteen hours after inoculation, bacteria were recovered from haemolymph of 15 melanized larvae. Serial dilutions were plated on GM17 supplemented with chloramphenicol. In this specific case of infection in the insect_A replica plating strategy was used, contrary to the mouse and urine experiments, because a tetracycline-resistant contaminating flora was present in the insect preventing us to select resolved bacteria on this antibiotic. After 72 h growth, colonies were replica-plated onto plates containing GM17 supplemented with chloramphenicol, erythromycin or tetracycline. After 24 h incubation at 37°C, plates were compared to identify erythromycin-sensitive and tetracycline-resistant colonies. Inserts cloned upstream from *cre* gene of these resolved cells were amplified by PCR and resulting amplicons were sequenced.

### Screening of the genomic library during exposure to urine

As explained above, genomic libraries were sub-cultured in presence of erythromycin to eliminate clones harboring a promoter active under *in vitro* conditions. *E. faecalis* V19 strain was tested during growth experiments in filter-sterilized urine. This showed that this strain was not able to double its population under these conditions (data not shown), contrary to strains MMH594 [44] and JH2.2 (personal communication). Thus, libraries were inoculated into urine without the supplementation of exogenous carbon sources or micronutrients for an overnight growth. Cells were then pelleted and resuspended into fresh urine for 3 hours at 37°C without aeration. Serial dilutions were then plated onto plates containing tetracycline. Colonies were then checked for their phenotypic switch by replica-plating on plates containing chloramphenicol, erythromycin or tetracycline. Cm^R^, Em^S^ and Tet^R^ colonies were then used as a template for PCR amplification of the insert cloned upstream from *cre*. Resulting amplicons were then sequenced.

*E. faecalis* V19 strain was also inoculated into filter-sterilized urine, as described previously. Three hours after exposure to fresh urine, RNAs were isolated and used for qRT-PCR experiments.

### Screening of the genomic library in mice infection models

Clones harboring active promoters under *in vitro* conditions were eliminated as explained above. The mouse bacteraemia model was then adapted from the method previously described by Hufnagel *et al*. [52], [53]. Eight 6–8 weeks-old female BALB/c mice were challenged by intravenous injection of Lox2 or Lox2ΔG libraries (5.10^8^ CFU per mouse) via the tail vein. Two, 4, 6 or 24 h after inoculation, mice were sacrificed and exsanguinated. Kidneys were removed and homogenized in 500 µl TSB. The murine peritonitis model was performed using 4–6 weeks-old female BALB/c mice that were injected i.p. with 1.7×10^10^ CFU of Lox2 or Lox2ΔG libraries in 200 µl sterile saline. Two, four and six hours after injection, mice were sacrificed, the peritoneal cavity was washed by injecting 5 ml sterile PBS, and bacteria in the resulting liquid were pelleted before being resuspended in 500 µl sterile PBS. After the peritoneal wash, rodents were also exsanguinated and kidneys were removed as described above. Serial dilutions of blood, homogenized kidneys and peritoneal wash were plated on GM17 containing tetracycline. After 72 h growth, colonies were replica plated onto plates containing GM17 with erythromycin, tetracycline or chloramphenicol. Plates were compared 24 h later to identify colonies that were sensitive to erythromycin and resistant to chloramphenicol and tetracycline. Colonies harboring such a phenotype were then used as a template to amplify insert cloned upstream from *cre* gene in pCre2. The resulting amplicons were subsequently sequenced.

### Resolution frequencies of the reporter cassette

To assess the validity of the *G. mellonella* R-IVET screen, the rate of *ermB* gene excision by the *ivi* promoters activity was determined by extracting pCre2 derivatives from *ivi* clones and using them to electroporate strain Lox2 or Lox2ΔG. The resolution rate after a about 15 generations *in vitro* growth was measured by plating serial dilution of culture and transferring 50 Cm^R^ colonies to GM17 plates containing chloramphenicol, erythromycin or tetracycline. The excision rate thus corresponds to the percentage of Em^S^, Cm^R^ and Tet^R^ colonies. Resolution frequencies after persistence in the animal host model were determined by injecting bacteria as described in animal experiments. Recovered bacteria in haemolyph were then plated onto GM17 plates supplemented with chloramphenicol. The excision rate was then determined as for *in vitro* growth.

### Construction of *E. faecalis* mutant strains

The RR02 mutant was constructed by Hancock and Perego [27]. The 377::Ery mutant was constructed using the insertional inactivation vector pOri19 as previously described [54]. Briefly, an internal fragment of *ef_0377* gene amplified from the chromosome of V583 strain was cloned into pOri19 vector. The recombinant plasmid was then electroporated into *E. faecalis* VE14412 and cells were plated on GM17 containing erythromycin and incubated at 30°C. Bacteria that received the plasmid were identified by colony PCR and then cultured at 42°C. Insertional mutants were identified by PCR. *E. faecalis* ΔTCS and Δ377 deletion mutants were constructed using the shuttle vector pMAD [55]. Upstream and downstream regions of the fragment to be deleted were cloned in the plasmid. After the transformation into the host, integration of the plasmid into the chromosome was selected during growth at a non-permissive temperature in the presence of erythromycin. The second cross-over event resulting in the deletion of the chosen region was induced by growth at the permissive temperature 30°C.

### Virulence assays

Virulence assays were performed in the insect model *Galleria mellonella* adapted from the method described by Park *et al*. [7]. Larvae were infected as explained above with *E. faecalis* overnight cultures in GM17. For each experiment, 15 larvae were used and tests were repeated at least three times. Larvae mortality was then monitored over 16 to 24 h post infection.

### Ethics statement

The study was approved by the Regierungspräsidium Freiburg, Az 35/9185.81/G-07/15.

## Supporting Information

Table S1 (0.02 MB XLS)

Table S2 (0.02 MB XLS)
